# Local control of striatal dopamine release

**DOI:** 10.3389/fnbeh.2014.00188

**Published:** 2014-05-23

**Authors:** Roger Cachope, Joseph F. Cheer

**Affiliations:** ^1^Department of Anatomy and Neurobiology, University of Maryland School of MedicineBaltimore, MD, USA; ^2^CHDI FoundationLos Angeles, CA, USA; ^3^Department of Psychiatry, University of Maryland School of MedicineBaltimore, MD, USA

**Keywords:** dopamine, acetylcholine, glutamate, striatum, optogenetics, axonal release, volume transmission

## Abstract

The mesolimbic and nigrostriatal dopamine (DA) systems play a key role in the physiology of reward seeking, motivation and motor control. Importantly, they are also involved in the pathophysiology of Parkinson’s and Huntington’s disease, schizophrenia and addiction. Control of DA release in the striatum is tightly linked to firing of DA neurons in the ventral tegmental area (VTA) and the substantia nigra (SN). However, local influences in the striatum affect release by exerting their action directly on axon terminals. For example, endogenous glutamatergic and cholinergic activity is sufficient to trigger striatal DA release independently of cell body firing. Recent developments involving genetic manipulation, pharmacological selectivity or selective stimulation have allowed for better characterization of these phenomena. Such termino-terminal forms of control of DA release transform considerably our understanding of the mesolimbic and nigrostriatal systems, and have strong implications as potential mechanisms to modify impaired control of DA release in the diseased brain. Here, we review these and related mechanisms and their implications in the physiology of ascending DA systems.

## Introduction: role of DA in motor and limbic function

Dopamine (DA) plays a critical role in the organization of reward-seeking behavior and motor responses (Joshua et al., 2009; Schultz, 2013). Through the mesolimbic and nigrostriatal DA systems, the forebrain receives dopaminergic input that modulates a range of functionally distinct structures, such as the basal ganglia and cerebral cortex (Björklund and Dunnett, 2007; Tritsch and Sabatini, 2012). The mesolimbic system is formed by dopaminergic neurons located in the VTA and their projections to the nucleus accumbens (NAc), cortex, amygdala and hippocampus, which participate in the configuration of reward-seeking behaviors (Björklund and Dunnett, 2007; Stuber et al., 2012; Nieh et al., 2013). The nigrostriatal system has its origin in the substantia nigra pars compacta (SNc) and projects preferentially to the dorsolateral domains of the striatum, having a more defined role in the organization of motor plans (Groenewegen, 2003; DeLong and Wichmann, 2007). Such functional distinction at the level of the striatum seems to have structural and molecular correlates on DA neurons from the SNc (Henny et al., 2012; Schiemann et al., 2012). Additional to these functional implications, dopaminergic transmission is compromised in a variety of neurological conditions such as schizophrenia, Huntington’s and Parkinson’s disease, drug addiction and obsessive-compulsive disorder, among others (DeLong and Wichmann, 2007; Money and Stanwood, 2013).

The striatum is the main input nucleus of the basal ganglia, and DA modulates how this input is processed (Calabresi et al., 1997; Centonze et al., 2001; Tritsch and Sabatini, 2012). However, in contrast to the traditional view of inter-neuronal chemical excitatory synaptic transmission in which structural and functional specializations are observed at the postsynaptic domains, striatal dopaminergic transmission does not always require such level of postsynaptic structural specialization (Rice and Cragg, 2008; Fuxe et al., 2012). Instead, release occurs in a diffuse manner, DA receptors are extrasynaptic and ultrastructural studies on the extension and density of DA neuron axonal arborization in the striatum point to broad, intricate projections that cover vast areas (Pickel et al., 1981; Smith et al., 1994; Moss and Bolam, 2008; Matsuda et al., 2009). This diffusely spread mode of transmission (in contrast to localized, highly spatially restricted communication), is termed “volume transmission”, and is a feature of a number of transmitters such as acetylcholine, norepinephrine, DA and serotonin (Taber and Hurley, 2014). Volume transmission of DA is, however, not exclusive to the striatum and it has its own particularities through different areas (Rice and Cragg, 2008; Fuxe et al., 2012; Martin and Spühler, 2013; Taber and Hurley, 2014). DA as a volume transmitter in the striatum is thought to exert a widespread modulatory influence on excitatory—glutamatergic—transmission arriving from the cortex, basolateral amygdala (BLA), and ventral hippocampus (vHipp; Britt et al., 2012); or on inhibitory—GABAergic—transmission incoming from areas such as VTA (Van Bockstaele and Pickel, 1995) and ventral pallidum (Churchill and Kalivas, 1994).

DA modulation of incoming transmission to the striatum plays a key role in the functional expression of reward-seeking behaviors and motor control. Such functions exhibit some stratification within the striatum (Threlfell and Cragg, 2011). For example, dorso-medial and dorso-lateral areas are predominantly involved in motor control, while ventro-medial segments are mostly involved in the expression of reward processing, motivation and salience (Groenewegen, 2003; Voorn et al., 2004; Kreitzer and Berke, 2011; Stuber et al., 2012). Concurrently, cortico-striatal projections also exhibit a stratified distribution in which the motor and cingulate cortices form the primary input to the dorso-lateral striatum, while prefrontal and prelimbic cortices project mainly to ventro-medial areas of the striatum (Voorn et al., 2004). Phenomena responsible for regulation of striatal DA release can be VTA/SNc driven, or locally acting, at the striatal level. This latter possibility has long been reported, still attracts considerable attention in terms of mechanistic characterization (Cachope et al., 2012; Threlfell et al., 2012) and is considered as an opportunity for functionally-segregated intervention (Threlfell and Cragg, 2011).

## Multiplicity of mechanisms in the control of dopamine release

Through what are now seminal papers, Wolfram Schultz et al. demonstrated that firing of DA neurons in the midbrain increases in response to rewarding stimuli in non-human primates (Schultz et al., 1997; Schultz, 1998), while functional imaging studies in humans point to a similar increase in cellular activity (D’Ardenne et al., 2008), suggesting correspondence with Schultz’s group reports. Interestingly, it was recently described that VTA GABAergic neurons also encode reward expectation (Cohen et al., 2012). Recordings of DA neurons from the VTA or SNc areas in rodents exhibit slow, tonic firing rates that periodically switch to a high frequency events (Grace and Bunney, 1984a, b). Thus, low levels of DA release have been correlated with low frequency firing rate of DA neurons, while corresponding enhancement in striatal DA release occurs in response to high frequency firing rates (Kawagoe et al., 1992). These findings have sculpted the traditional view of striatal DA release being determined by the rate of neuronal firing of the DA neuron somatas located in either VTA or SNc. However, besides this dominant mechanism of control of DA release, local factors such as reuptake, autoreceptor-dependent modulation, and termino-terminal control exist and are recognized to play a prominent role, independently of VTA/SNc firing rate.

DA neurons projecting to the striatum establish prominent axonal trees at their destination. The volume transmission feature of striatal DA implies that a considerable amount of control is required in terms of uptake and/or negative feedback on future release events. In reaching this goal, two key mechanisms are DA transporter activity (DAT) and D2-like presynaptic autoreceptor activity. DAT activity is thought to limit the radius of DA activity (Rice and Cragg, 2008) and, by doing so, restricts activation of DA receptors (reviewed in Rice et al., 2011). In a similar manner, it is known that blockade of D2-like DA receptors in slices leads to increased DA release in response to repetitive electrical stimulation (Limberger et al., 1991; Patel et al., 1992). This effect, however, is not manifest when single pulse stimulation is used (Limberger et al., 1991; Patel et al., 1992), suggesting that there is not sufficient DA tone elicited by a single pulse to be displaced by the antagonist. Importantly, changes in D2 receptor levels and their subsequent activation are thought to play a prominent role in several neurological conditions in which DA levels are altered (Ford, 2014).

## Local striatal control of dopamine release

### Glutamatergic transmission

Excitatory glutamatergic activity in the striatum originates mainly from frontal cortex, midline and intralaminar thalamus, basal amygdala, and hippocampus (reviewed in Sesack and Grace, 2010; Stuber et al., 2012). Additionally, DA terminals release glutamate (Sulzer et al., 1998; Joyce and Rayport, 2000; Sulzer and Rayport, 2000; Chuhma et al., 2004; Dal Bo et al., 2004; Chuhma et al., 2009; Hnasko et al., 2010), and this has recently been demonstrated by way of selective optogenetic stimulation of DA terminals (Stuber et al., 2010). However, this last report demonstrates that such possibility exists only in DA terminals that reach the NAc, not the dorsal striatum. Still, some debate prevails as to this feature not being present in the adult brain (Bérubé-Carrière et al., 2009; Moss et al., 2011), or being as widespread as initially thought (Stuber et al., 2010; for a review, see Broussard, 2012).

Evidence on the potential role of glutamate as a form of local control of DA release in the striatum has long been reported (Imperato et al., 1990; Cheramy et al., 1991; Krebs et al., 1991; Desce et al., 1992) and both ionotropic and metabotropic glutamate receptors (iGluR; mGluR, respectively) have been implicated. However, most of the initial studies were performed *in vivo* using brain microdialysis as the measuring technique to assess DA levels as well as for local administration of glutamate receptor ligands. Such findings were of course influenced by slow temporal resolution and the effects of the ligand in a complex circuit, among other factors, making a mechanistic interpretation difficult. *In vitro* experimental designs, on the other hand, allowed for more direct mechanistic description while still not directly addressing whether results were equivalent to intact-tissue conditions. These distinct experimental conditions might account for what, at the time, were apparent contradictory results. Initial *in vitro* explorations in slices and synaptosomes accounted not only for glutamate, but for a range of neurotransmitters that could affect striatal DA release locally, including acetylcholine, GABA, glycine and opiates (reviewed in Chesselet, 1984). However, further *in vivo* experiments in freely moving rats were still non conclusive; i.e., activation of AMPA receptors by exogenous ligands led to a decrease in DA release, while an increase was evident only in response to the application of NMDA receptor ligands at high concentrations (Imperato et al., 1990). Blocking uptake of endogenous release, in turn, elevated DA release in a way that was sensitive to the application of either NMDA or AMPA antagonists, suggesting the involvement of both receptor types in that response (Segovia et al., 1997). Similarly, electrical stimulation of the prefrontal cortex, a putative glutamatergic input to striatum, as well as local application of kainate or NMDA increased DA release (Cheramy et al., 1991; Krebs et al., 1991). Development of electrochemical techniques, however, greatly contributed to the clarification of these mechanisms. The use of fast-scan cyclic voltammetry (FSCV) for the detection of DA *in vitro* allowed for better temporal resolution which was less influenced by circuit adaptive responses in the mid-term scale (minutes), which could potentially influence DA readout. Under those conditions, bath application of kainate, AMPA or NMDA elicited inhibition of DA release (Wu et al., 2000; Kulagina et al., 2001; Avshalumov et al., 2003). Moreover, electron microscopy studies were not able to demonstrate labeling of iGluRs in striatal DA terminals (Bernard et al., 1997; Bernard and Bolam, 1998). The lack of expression of iGluRs on DA terminals suggests that iGluR-mediated modulation of DA release relates to a more complex process; which may underlie interactions between multiple cellular types and/or chemical mediators. This issue, raised and investigated by Rice’s group led to the identification of H_2_O_2_ as a key molecule in the iGluR-mediated decrease of DA release (Avshalumov et al., 2000, 2003, 2008; Avshalumov and Rice, 2003). This model describes how glutamatergic activity on ionotropic receptors in medium spiny neurons (MSNs) triggers production and release of H_2_O_2_, which in turn diffuses to adjacent DA terminals and promotes opening of K_ATP_ channels leading to reduction of DA release (Avshalumov et al., 2008).

In contrast to iGluRs, labeling of mGluRs has been reported in presynaptic profiles identified as DA axons (Paquet and Smith, 2003). Moreover, blocking glutamate uptake, or high-frequency stimulation of the cortico-striatal pathway modulates DA release, in a mGluR-dependent fashion followed by modulation of Ca^++^-activated potassium channels (Zhang and Sulzer, 2003). Altogether, the existent evidence points to mGluR-mediated direct action on DA terminals, and a second MSN-mediated mechanism involving iGluR-H_2_O_2_ signaling.

### Cholinergic transmission

In contrast to striatal glutamatergic activity, which originates mainly from inputs to the striatum, sources of striatal acetylcholine release are mostly from cholinergic interneurons (CINs) that account for about 2–5% of all striatal neurons (Descarries et al., 1997; Descarries and Mechawar, 2000). Additional to CINs, a recent report shows that brainstem-based cholinergic neurons send terminals to the striatum in a topographic fashion with their origin (Dautan et al., 2014). In spite of their low numbers, CINs establish prominent and intricate axonal projections that configure an extensive planar neurotransmission system (Descarries et al., 1997; Descarries and Mechawar, 2000). Electrophysiological characterization shows that CINs are tonically active neurons that fire at a relatively low rate of about 5–10 Hz (Wilson et al., 1990; Aosaki et al., 1995). This rate, however, as in the case of DA neurons, encodes behaviorally relevant reward-related events (Apicella et al., 1991, 2011; Aosaki et al., 1994, 1995; Shimo and Hikosaka, 2001; Morris et al., 2004).

Target receptors of cholinergic activity in the striatum are both of nicotinic and muscarinic types (nAChR and mAChR, respectively). While mAChRs are seven trans-membrane domain G-protein coupled receptors, nAChRs consist of five subunits arranged as homomers or heteromers that, in mammals, are formed by subfamilies II (α7) and III (α2-6, β2-4) (Le Novère et al., 2002). Particularly, striatal DA axons express a high density of α4, α5, α6, β2 and β3 subunits in an arrangement of two αβ pairs that could be α4-β2 and/or α6-β2 and/or α4-β4, plus a fifth subunit that can be α5 or β3 (Champtiaux et al., 2003; reviewed in Threlfell and Cragg, 2011). Additionally, the β2 subunit is expressed on striatal DA axons (Jones et al., 2001) and is included in all nAChRs at these terminals. This characterization is functionally relevant because some segregation exists in which predominance of different α subunits occurs between dorso-lateral striatum and the NAc. More specifically, a significant amount of work has shown that α4(non-α6)-nAChRs play a prominent role in dorsal striatum, while α4α6-nAChRs are dominant in NAc (Exley et al., 2008, 2011, 2012). Given the distinct functional role of the dorsolateral and the ventromedial striatum, it has been proposed that such differences could be taken into account as a substrate for region-specific intervention (Threlfell and Cragg, 2011).

mAChRs, in turn, are classified in two groups according to their coupling to either G_s_ (M_1_, M_3_, M_5_) or G_i_ (M_2_, M_4_) subunits of G proteins, with M_2_ and M_4_ predominantly expressed in CINs (Yan and Surmeier, 1996). In a similar way to what has been described for nAChRs, mAChRs exhibit some dorso-ventral gradient in their ability to regulate DA release. While M_2_/M_4_ receptors are necessary for such regulation in the dorsal striatum, M4 is prevalent in the NAc (Threlfell et al., 2010). Additionally, expression of M_5_ receptors has been reported in nigrostriatal DA neurons, although their pattern of expression on striatal DA terminals and subsequent potential role in local control of DA release remains unclear (reviewed in Threlfell and Cragg, 2011; Zhang and Sulzer, 2012).

Involvement of presynaptic cholinergic receptors on DA regulation was inferred early, mainly from experiments describing increase of DA release in response to AChR activation in slices or synaptosomes (Giorguieff et al., 1976, 1977; Wonnacott et al., 1989; Rapier et al., 1990). In a similar way to what occurred with the characterization of glutamatergic-dependent DA modulation, transition to electrochemical methods to quantify DA allowed for a better temporal resolution. Importantly, FSCV has been critical in determining a high dependence of DA release on stimulation frequency under the effect of nicotine. More specifically, in a striatal slice, the maximum peak of DA release does not change significantly through different frequencies (5, 10, 25, 50 Hz) of electrical stimulation. However, in the presence of nicotine or the nAChR antagonist mecamylamine, DA release at low frequencies is decreased, while at high frequencies release is enhanced (Rice and Cragg, 2004).

While electrical stimulation combined with pharmacological and genetic manipulations have produced a wealth of information on cholinergic control of DA release (Giorguieff et al., 1977; Cheramy et al., 1991; Krebs et al., 1991; Desce et al., 1992; Tremblay et al., 1992; Chéramy et al., 1996; Schmitz et al., 2003; Zhang and Sulzer, 2003, 2012; Exley and Cragg, 2008; Exley et al., 2011; reviewed by Cragg, 2006; Rice et al., 2011; Threlfell and Cragg, 2011; Zhang and Sulzer, 2012), the advent of optogenetics offered the previously unseen possibility of selective control of CINs. This would allow inducing AChR activation by means of endogenous release of ACh, obtained by selective stimulation of striatal CINs. Taking advantage of *in vitro* slices, electrochemistry, and optogenetics, both the laboratory of Stephanie Cragg (Threlfell et al., 2012) and ours (Cachope et al., 2012) were interested in characterizing changes in striatal DA levels in response to endogenous AChR activation. Interestingly, both research groups were advancing on characterizing similar phenomena in functionally different striatal areas, showing how endogenous release of ACh directly triggers DA release in the dorsal striatum and the NAc, respectively. Additionally to demonstrating how selective activation of CINs is enough to trigger DA release in striatum and NAc, respectively, both reports confirmed the role of nAChRs and mAChRs in modulating such output. Moreover, Cragg’s report very nicely unveiled circuital mechanisms by which thalamic input synchronizes CIN firing, subsequently promoting DA release (Threlfell et al., 2012). Our experiments focused instead on the possibility of CINs-triggered DA release *in vivo* (Cachope et al., 2012). Consistently with the *in vitro* data, optogenetic stimulation of CINs in the anesthetized mouse was sufficient to trigger elevation of DA concentrations in the NAc. Also, following Sabatini’s group’s report regarding the ability of CINs to evoke glutamatergic responses (Higley et al., 2011), we showed that ACh-evoked DA release is sensitive to AMPA blockers (Cachope et al., 2012).

In the case of mAChRs, they can also locally modulate DA release in the striatum. *In vitro* experiments with FSCV show how a wide range mAChR antagonist (oxotremorine) decreases DA release evoked by single pulse electrical stimulation, but enhances DA levels in response to train stimulation (Threlfell et al., 2010). A similar effect was observed using selective optogenetic stimulation, in which single pulse optical stimulation did not affect DA release, but instead 5 and 10 Hz stimulation enhanced DA release under application of the mAChR antagonist scopolamine (Cachope et al., 2012; Threlfell et al., 2012).

**Figure 1:**
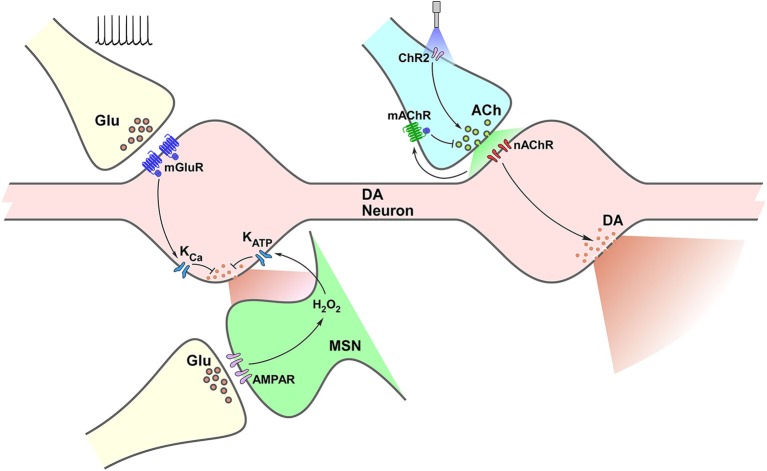
**Termino-terminal control of dopamine (DA) release in the striatum**. Model diagram of glutamatergic (left side of graph) and cholinergic (right side of graph) local influences on striatal DA release. Electrically-evoked glutamate release activates mGluRs located on dopaminergic varicosities increasing Ca^++^-sensitive K channels (K_Ca_) conductance, which leads to reduction of DA release. Activation of iGluRs on MSNs elevates production of H_2_O_2_, which diffuses to DA varicosities enhancing ATP-sensitive K channels (K_ATP_) conductance reducing DA release. Optogenetic selective activation of cholinergic interneurons (CINs) through channelrhodopsin (ChR2) triggers ACh release, increasing nAChR activation on DA varicosities, triggering DA release. Activation of mAChRs on cholinergic terminals decreases ACh release and further nAChR activation, which would result in decreased DA release.

A complex interaction between diverse neurotransmission and neuromodulatory systems takes place in the control of striatal DA release. Although we have focused on the effect of glutamatergic and cholinergic systems, a handful other receptors have been identified as able to alter striatal DA levels; including GABA, cannabinoid, purinergic and opioid. Interestingly (and, up to some point expected), the possibilities for diversity on this local control are dependent on the type of receptor, not just the type of transmitter/modulator being released. Both in the case of glutamate and acetylcholine, different receptors lead to distinct and even opposite effects. As illustrated in Figure 1, mGluR activation on DA terminals and iGluR activation on MSNs result both in modulation of K conductances decreasing DA release. In contrast, activation of nAChRs on DA terminals leads to increased DA release, while activation of mAChR autoreceptors expectedly result in decreased DA release. More importantly, all these results demonstrate that firing rate at the VTA and SNc does not entirely determine striatal DA output, leaving enough room for control mechanisms driven by input from other areas (glutamatergic), as well as by interneurons (cholinergic), which might exert considerable impact on it.

## Concluding remarks

The role of DA in essential behaviors such as reward-seeking, motivation and motor control has been extensively studied. Regulation of DA release at both the dorso-lateral striatum and the NAc is considered to be mainly the consequence of changes in firing rate at the level of DA somata in the SNc and the VTA, correspondingly. Local control at the level of the striatum has been traditionally linked to DA reuptake and to feedback control on DA release through activation of D2 autoreceptors. However, reports on termino-terminal control of DA release, although scarce decades ago provided key findings in understanding a more complex control system than the one defined just by firing rate at DA neuronal somata. To date, the influence of non-DA striatal terminals on striatal DA release has been explored in a variety of experimental conditions, including synaptosomes, *in vitro* slices and *in vivo* preparations. Not only pharmacological, but genetic, optogenetic, electrophysiological and electrochemical strategies have been used to unveil the localization, role, extent and functional impact of such local influences. Glutamatergic and cholinergic systems have attracted the most attention so far. Still, although highly characterized in terms of types of receptors and neurotransmitters involved, there is not enough evidence on the functional impact of these forms of regulation in the behavioral setting. CINs modify their firing rate in animals subject to behavioral tasks encoding reward delivery as a decrease in firing rate, following a mild increase in frequency of firing (Apicella et al., 1991, 2011; Aosaki et al., 1994; Shimo and Hikosaka, 2001; Morris et al., 2004). Also, a recent report shows a differential role of DA neurons modulating CINs firing in dorsal striatum and NAc (Chuhma et al., 2014). However, there is no clarity as to how prominent all those interactions are in terms of their ability to affect DA release, and even less is known about the role of such variations, if they might impact behavior, or if DA transmission is otherwise still VTA- and SNc-driven.

One of the main strategies to fully develop yet is the potential of targeting these modulation systems to affect striatal DA release in conditions such as Parkinson’s disease, schizophrenia, addiction, Huntington’s disease, in which DA levels have been reported to be altered. As already outlined by Threlfell and Cragg (2011), modulating the striatal cholinergic system through subunit-specific modulation of nAChR and mAChR promises to be a useful approach. Temporal dynamics are a critical feature of inter-neuronal transmission. Behavioral events have, for example, phasic changes in striatal DA levels as correlates in the limbic and motor areas (O’Neill and Fillenz, 1985; Schultz, 2007a, b; Joshua et al., 2009). A significant proportion of therapeutic strategies are based on ligands that exert a sustained effect on neurotransmitter receptors, cancelling such changes over time. While DA neuron somata drive phasic changes in DA release, termino-terminal control might be seen as a mechanism that allows for fine regulation over that main drive, still preserving most of the temporal dynamics.

## Conflict of interest statement

The authors declare that the research was conducted in the absence of any commercial or financial relationships that could be construed as a potential conflict of interest.

## References

[B1] Aosaki T. Kimura M. Graybiel A. M. (1995). Temporal and spatial characteristics of tonically active neurons of the primate’s striatum. J. Neurophysiol. 73, 1234–1252 7608768 10.1152/jn.1995.73.3.1234

[B2] Aosaki T. Tsubokawa H. Ishida A. Watanabe K. Graybiel A. Kimura M. (1994). Responses of tonically active neurons in the primate’s striatum undergo systematic changes during behavioral sensorimotor conditioning. J. Neurosci. 14, 3969–3984 8207500 10.1523/JNEUROSCI.14-06-03969.1994 PMC6576948

[B3] Apicella P. Ravel S. Deffains M. Legallet E. (2011). The role of striatal tonically active neurons in reward prediction error signaling during instrumental task performance. J. Neurosci. 31, 1507–1515 10.1523/jneurosci.4880-10.2011 21273435 PMC6623604

[B4] Apicella P. Scarnati E. Schultz W. (1991). Tonically discharging neurons of monkey striatum respond to preparatory and rewarding stimuli. Exp. Brain Res. 84, 672–675 10.1007/bf00230981 1864338

[B5] Avshalumov M. V. Chen B. T. Marshall S. P. Peña D. M. Rice M. E. (2003). Glutamate-dependent inhibition of dopamine release in striatum is mediated by a new diffusible messenger, H2O2. J. Neurosci. 23, 2744–2750 10.1073/pnas.1834314100 12684460 PMC6742066

[B6] Avshalumov M. V. Chen B. T. Rice M. E. (2000). Mechanisms underlying H(2)O(2)-mediated inhibition of synaptic transmission in rat hippocampal slices. Brain Res. 882, 86–94 10.1016/s0006-8993(00)02835-3 11056187

[B7] Avshalumov M. Patel J. Rice M. (2008). AMPA receptor-dependent H 2 O 2 generation in striatal spiny neurons, but not dopamine axons: one source of a retrograde signal that can inhibit dopamine release. J. Neurophysiol. 100, 1590–1601 10.1152/jn.90548.2008 18632893 PMC2544473

[B8] Avshalumov M. Rice M. (2003). Activation of ATP-sensitive K+ (KATP) channels by H2O2 underlies glutamate-dependent inhibition of striatal dopamine release. Proc. Natl. Acad. Sci. U S A 100, 11729–11734 10.1073/pnas.1834314100 13679582 PMC208826

[B9] Bernard V. Bolam J. P. (1998). Subcellular and subsynaptic distribution of the NR1 subunit of the NMDA receptor in the neostriatum and globus pallidus of the rat: co-localization at synapses with the GluR2/3 subunit of the AMPA receptor. Eur. J. Neurosci. 10, 3721–3736 10.1046/j.1460-9568.1998.00380.x 9875351

[B10] Bernard V. Somogyi P. Bolam J. P. (1997). Cellular, subcellular and subsynaptic distribution of AMPA-type glutamate receptor subunits in the neostriatum of the rat. J. Neurosci. 17, 819–833 8987803 10.1523/JNEUROSCI.17-02-00819.1997 PMC6573249

[B11] Bérubé-Carrière N. Riad M. Dal Bo G. Lévesque D. Trudeau L.-E. Descarries L. (2009). The dual dopamine-glutamate phenotype of growing mesencephalic neurons regresses in mature rat brain. J. Comp. Neurol. 517, 873–891 10.1002/cne.22194 19844994

[B12] Björklund A. Dunnett S. B. (2007). Dopamine neuron systems in the brain: an update. Trends Neurosci. 30, 194–202 10.1016/j.tins.2007.03.006 17408759

[B13] Britt J. P. Benaliouad F. McDevitt R. A. Stuber G. D. Wise R. A. Bonci A. (2012). Synaptic and behavioral profile of multiple glutamatergic inputs to the nucleus accumbens. Neuron 76, 790–803 10.1016/j.neuron.2012.09.040 23177963 PMC3607383

[B14] Broussard J. I. (2012). Co-transmission of dopamine and glutamate. J. Gen. Physiol. 139, 93–96 10.1085/jgp.201110659 22200950 PMC3250102

[B15] Cachope R. Mateo Y. Mathur B. N. Irving J. Wang H. Morales M. (2012). Selective activation of cholinergic interneurons enhances accumbal phasic dopamine release: setting the tone for reward processing. Cell Rep. 2, 33–41 10.1016/j.celrep.2012.05.011 22840394 PMC3408582

[B16] Calabresi P. Pisani A. Centonze D. Bernardi G. (1997). Synaptic plasticity and physiological interactions between dopamine and glutamate in the striatum. Neurosci. Biobehav. Rev. 21, 519–523 10.1016/s0149-7634(96)00029-2 9195611

[B17] Centonze D. Picconi B. Gubellini P. Bernardi G. Calabresi P. (2001). Dopaminergic control of synaptic plasticity in the dorsal striatum. Eur. J. Neurosci. 13, 1071–1077 10.1046/j.0953-816x.2001.01485.x 11285003

[B18] Champtiaux N. Gotti C. Cordero-Erausquin M. David D. J. Przybylski C. Léna C. (2003). Subunit composition of functional nicotinic receptors in dopaminergic neurons investigated with knock-out mice. J. Neurosci. 23, 7820–7829 12944511 10.1523/JNEUROSCI.23-21-07820.2003 PMC6740613

[B19] Chéramy A. Godeheu G. L’Hirondel M. Glowinski J. (1996). Cooperative contributions of cholinergic and NMDA receptors in the presynaptic control of dopamine release from synaptosomes of the rat striatum. J. Pharmacol. Exp. Ther. 276, 616–625 8632329

[B20] Cheramy A. Kemel M. L. Gauchy C. Desce J. M. J. Galli T. Barbeito L. (1991). Role of excitatory amino acids in the direct and indirect presynaptic regulation of dopamine release from nerve terminals of nigrostriatal dopaminergic neurons. Amino Acids 1, 351–363 10.1007/bf00814004 24194175

[B21] Chesselet M. F. (1984). Presynaptic regulation of neurotransmitter release in the brain: facts and hypothesis. Neuroscience 12, 347–375 10.1016/0306-4522(84)90058-7 6146946

[B22] Chuhma N. Choi W. Y. Mingote S. Rayport S. (2009). Dopamine neuron glutamate cotransmission: frequency-dependent modulation in the mesoventromedial projection. Neuroscience 164, 1068–1083 10.1016/j.neuroscience.2009.08.057 19729052 PMC2783216

[B23] Chuhma N. Mingote S. Moore H. Rayport S. (2014). Dopamine neurons control striatal cholinergic neurons via regionally heterogeneous dopamine and glutamate signaling. Neuron 81, 901–912 10.1016/j.neuron.2013.12.027 24559678 PMC3933825

[B24] Chuhma N. Zhang H. Masson J. Zhuang X. Sulzer D. Hen R. (2004). Dopamine neurons mediate a fast excitatory signal via their glutamatergic synapses. J. Neurosci. 24, 972–981 10.1523/jneurosci.4317-03.2004 14749442 PMC6729804

[B25] Churchill L. Kalivas P. W. (1994). A topographically organized gamma-aminobutyric acid projection from the ventral pallidum to the nucleus accumbens in the rat. J. Comp. Neurol. 345, 579–595 10.1002/cne.903450408 7962701

[B26] Cohen J. Y. Haesler S. Vong L. Lowell B. B. Uchida N. (2012). Neuron-type-specific signals for reward and punishment in the ventral tegmental area. Nature 482, 85–88 10.1038/nature10754 22258508 PMC3271183

[B27] Cragg S. J. (2006). Meaningful silences: how dopamine listens to the ACh pause. Trends Neurosci. 29, 125–131 10.1016/j.tins.2006.01.003 16443285

[B28] D’Ardenne K. McClure S. M. Nystrom L. E. Cohen J. D. (2008). BOLD responses reflecting dopaminergic signals in the human ventral tegmental area. Science 319, 1264–1267 10.1126/science.1150605 18309087

[B29] Dal Bo G. St-Gelais F. Danik M. Williams S. Cotton M. Trudeau L.-E. (2004). Dopamine neurons in culture express VGLUT2 explaining their capacity to release glutamate at synapses in addition to dopamine. J. Neurochem. 88, 1398–1405 10.1046/j.1471-4159.2003.02277.x 15009640

[B30] Dautan D. Huerta-Ocampo I. Witten I. B. Deisseroth K. Bolam J. P. Gerdjikov T. (2014). A major external source of cholinergic innervation of the striatum and nucleus accumbens originates in the brainstem. J. Neurosci. 34, 4509–4518 10.1523/JNEUROSCI.5071-13.2014 24671996 PMC3965779

[B31] DeLong M. R. Wichmann T. (2007). Circuits and circuit disorders of the basal ganglia. Arch. Neurol. 64, 20–24 10.1001/archneur.64.1.20 17210805

[B32] Descarries L. Gisiger V. Steriade M. (1997). Diffuse transmission by acetylcholine in the CNS. Prog. Neurobiol. 53, 603–625 10.1016/s0301-0082(97)00050-6 9421837

[B33] Descarries L. Mechawar N. (2000). Ultrastructural evidence for diffuse transmission by monoamine and acetylcholine neurons of the central nervous system. Prog. Brain Res. 125, 27–47 10.1016/s0079-6123(00)25005-x 11098652

[B34] Desce J. M. Godeheu G. Galli T. Artaud F. Chéramy A. Glowinski J. (1992). L-glutamate-evoked release of dopamine from synaptosomes of the rat striatum: involvement of AMPA and N-methyl-D-aspartate receptors. Neuroscience 47, 333–339 10.1016/0306-4522(92)90249-2 1379352

[B35] Exley R. Clements M. A. Hartung H. McIntosh J. M. Cragg S. J. (2008). Alpha6-containing nicotinic acetylcholine receptors dominate the nicotine control of dopamine neurotransmission in nucleus accumbens. Neuropsychopharmacology 33, 2158–2166 10.1038/sj.npp.1301617 18033235

[B36] Exley R. Cragg S. J. (2008). Presynaptic nicotinic receptors: a dynamic angic filter of striatal dopamine neurotransmission. Br. J. Pharmacol. 153(Suppl.), S283–S297 10.1038/sj.bjp.0707510 18037926 PMC2268048

[B37] Exley R. Maubourguet N. David V. Eddine R. Evrard A. Pons S. (2011). Distinct contributions of nicotinic acetylcholine receptor subunit alpha4 and subunit alpha6 to the reinforcing effects of nicotine. Proc. Natl. Acad. Sci. U S A 108, 7577–7582 10.1073/pnas.1103000108 21502501 PMC3088627

[B38] Exley R. McIntosh J. M. Marks M. J. Maskos U. Cragg S. J. (2012). Striatal α5 nicotinic receptor subunit regulates dopamine transmission in dorsal striatum. J. Neurosci. 32, 2352–2356 10.1523/JNEUROSCI.4985-11.2012 22396410 PMC3742968

[B39] Ford C. P. (2014). The role of D2-autoreceptors in regulating dopamine neuron activity and transmission. Neuroscience [Epub ahead of print]. 10.1016/j.neuroscience.2014.01.025 24463000 PMC4108583

[B40] Fuxe K. Borroto-Escuela D. O. Romero-Fernandez W. Diaz-Cabiale Z. Rivera A. Ferraro L. (2012). Extrasynaptic neurotransmission in the modulation of brain function. Focus on the striatal neuronal-glial networks. Front. Physiol. 3:136 10.3389/fphys.2012.00136 22675301 PMC3366473

[B41] Giorguieff M. F. Le Floc’h M. L. Glowinski J. Besson M. J. (1977). Involvement of cholinergic presynaptic receptors of nicotinic and muscarinic types in the control of the spontaneous release of dopamine from striatal dopaminergic terminals in the rat. J. Pharmacol. Exp. Ther. 200, 535–544 850127

[B42] Giorguieff M. F. Le Floc’h M. L. Westfall T. C. Glowinski J. Besson M. J. (1976). Nicotinic effect of acetylcholine on the release of newly synthesized [3H]dopamine in rat striatal slices and cat caudate nucleus. Brain Res. 106, 117–131 10.1016/0006-8993(76)90077-9 1268701

[B43] Grace A. Bunney B. (1984a). The control of firing pattern in nigral dopamine neurons: single spike firing. J. Neurosci. 4, 2866–2876 6150070 10.1523/JNEUROSCI.04-11-02866.1984 PMC6564731

[B44] Grace A. Bunney B. (1984b). The control of firing pattern in nigral dopamine neurons: burst firing. J. Neurosci. 4, 2877–2890 6150071 10.1523/JNEUROSCI.04-11-02877.1984 PMC6564720

[B45] Groenewegen H. J. (2003). The basal ganglia and motor control. Neural Plast. 10, 107–120 10.1155/np.2003.107 14640312 PMC2565420

[B46] Henny P. Brown M. T. C. Northrop A. Faunes M. Ungless M. A. Magill P. J. (2012). Structural correlates of heterogeneous in vivo activity of midbrain dopaminergic neurons. Nat. Neurosci. 15, 613–619 10.1038/nn.3048 22327472 PMC4242968

[B47] Higley M. J. Gittis A. H. Oldenburg I. A. Balthasar N. Seal R. P. Edwards R. H. (2011). Cholinergic interneurons mediate fast VGluT3-dependent glutamatergic transmission in the striatum. PLoS One 6:e19155 10.1371/journal.pone.0019155 21544206 PMC3081336

[B48] Hnasko T. S. Chuhma N. Zhang H. Goh G. Y. Sulzer D. Palmiter R. D. (2010). Vesicular glutamate transport promotes dopamine storage and glutamate corelease in vivo. Neuron 65, 643–656 10.1016/j.neuron.2010.02.012 20223200 PMC2846457

[B49] Imperato A. Honoré T. Jensen L. H. (1990). Dopamine release in the nucleus caudatus and in the nucleus accumbens is under glutamatergic control through non-NMDA receptors: a study in freely-moving rats. Brain Res. 530, 223–228 10.1016/0006-8993(90)91286-p 2176114

[B50] Jones I. W. Bolam J. P. Wonnacott S. (2001). Presynaptic localisation of the nicotinic acetylcholine receptor beta2 subunit immunoreactivity in rat nigrostriatal dopaminergic neurones. J. Comp. Neurol. 439, 235–247 10.1002/cne.1345 11596051

[B51] Joshua M. Adler A. Bergman H. (2009). The dynamics of dopamine in control of motor behavior. Curr. Opin. Neurobiol. 19, 615–620 10.1016/j.conb.2009.10.001 19896833

[B52] Joyce M. P. Rayport S. (2000). Mesoaccumbens dopamine neuron synapses reconstructed in vitro are glutamatergic. Neuroscience 99, 445–456 10.1016/s0306-4522(00)00219-0 11029537

[B53] Kawagoe K. T. Garris P. A. Wiedemann D. J. Wightman R. M. (1992). Regulation of transient dopamine concentration gradients in the microenvironment surrounding nerve terminals in the rat striatum. Neuroscience 51, 55–64 10.1016/0306-4522(92)90470-m 1465186

[B54] Krebs M. O. Desce J. M. Kemel M. L. Gauchy C. Godeheu G. Cheramy A. (1991). Glutamatergic control of dopamine release in the rat striatum: evidence for presynaptic N-Methyl-D-aspartate receptors on dopaminergic nerve terminals. J. Neurochem. 56, 81–85 10.1111/j.1471-4159.1991.tb02565.x 1824785

[B55] Kreitzer A. C. Berke J. D. (2011). Investigating striatal function through cell-type-specific manipulations. Neuroscience 198, 19–26 10.1016/j.neuroscience.2011.08.018 21867745 PMC3221791

[B56] Kulagina N. V. Zigmond M. J. Michael A. C. (2001). Glutamate regulates the spontaneous and evoked release of dopamine in the rat striatum. Neuroscience 102, 121–128 10.1016/s0306-4522(00)00480-2 11226675

[B57] Le Novère N. Corringer P.-J. Changeux J.-P. (2002). The diversity of subunit composition in nAChRs: evolutionary origins, physiologic and pharmacologic consequences. J. Neurobiol. 53, 447–456 10.1002/neu.10153 12436412

[B58] Limberger N. Trout S. J. Kruk Z. L. Starke K. (1991). “Real time” measurement of endogenous dopamine release during short trains of pulses in slices of rat neostriatum and nucleus accumbens: role of autoinhibition. Naunyn Schmiedebergs Arch. Pharmacol. 344, 623–629 1775195 10.1007/BF00174745

[B59] Martin K. A. C. Spühler I. A. (2013). The fine structure of the dopaminergic innervation of area 10 of macaque prefrontal cortex. Eur. J. Neurosci. 37, 1061–1071 10.1111/ejn.12124 23331617

[B60] Matsuda W. Furuta T. Nakamura K. C. Hioki H. Fujiyama F. Arai R. (2009). Single nigrostriatal dopaminergic neurons form widely spread and highly dense axonal arborizations in the neostriatum. J. Neurosci. 29, 444–453 10.1523/jneurosci.4029-08.2009 19144844 PMC6664950

[B61] Money K. M. Stanwood G. D. (2013). Developmental origins of brain disorders: roles for dopamine. Front. Cell. Neurosci. 7:260 10.3389/fncel.2013.00260 24391541 PMC3867667

[B62] Morris G. Arkadir D. Nevet A. Vaadia E. Bergman H. (2004). Coincident but distinct messages of midbrain dopamine and striatal tonically active neurons. Neuron 43, 133–143 10.1016/j.neuron.2004.06.012 15233923

[B63] Moss J. Bolam J. P. (2008). A dopaminergic axon lattice in the striatum and its relationship with cortical and thalamic terminals. J. Neurosci. 28, 11221–11230 10.1523/jneurosci.2780-08.2008 18971464 PMC6671499

[B64] Moss J. Ungless M. A. Bolam J. P. (2011). Dopaminergic axons in different divisions of the adult rat striatal complex do not express vesicular glutamate transporters. Eur. J. Neurosci. 33, 1205–1211 10.1111/j.1460-9568.2011.07594.x 21375596

[B65] Nieh E. H. Kim S.-Y. Namburi P. Tye K. M. (2013). Optogenetic dissection of neural circuits underlying emotional valence and motivated behaviors. Brain Res. 1511, 73–92 10.1016/j.brainres.2012.11.001 23142759 PMC4099056

[B66] O’Neill R. D. Fillenz M. (1985). Simultaneous monitoring of dopamine release in rat frontal cortex, nucleus accumbens and striatum: effect of drugs, circadian changes and correlations with motor activity. Neuroscience 16, 49–55 10.1016/0306-4522(85)90046-6 3835502

[B67] Paquet M. Smith Y. (2003). Group I metabotropic glutamate receptors in the monkey striatum: subsynaptic association with glutamatergic and dopaminergic afferents. J. Neurosci. 23, 7659–7669 12930805 10.1523/JNEUROSCI.23-20-07659.2003 PMC6740746

[B68] Patel J. Trout S. J. Kruk Z. L. (1992). Regional differences in evoked dopamine efflux in brain slices of rat anterior and posterior caudate putamen. Naunyn Schmiedebergs Arch. Pharmacol. 346, 267–276 10.1007/bf00173539 1407013

[B69] Pickel V. M. Beckley S. C. Joh T. H. Reis D. J. (1981). Ultrastructural immunocytochemical localization of tyrosine hydroxylase in the neostriatum. Brain Res. 225, 373–385 10.1016/0006-8993(81)90843-x 6118197

[B70] Rapier C. Lunt G. G. Wonnacott S. (1990). Nicotinic modulation of [3H]dopamine release from striatal synaptosomes: pharmacological characterisation. J. Neurochem. 54, 937–945 10.1111/j.1471-4159.1990.tb02341.x 2303820

[B71] Rice M. E. Cragg S. J. (2008). Dopamine spillover after quantal release: rethinking dopamine transmission in the nigrostriatal pathway. Brain Res. Rev. 58, 303–313 10.1016/j.brainresrev.2008.02.004 18433875 PMC2879278

[B72] Rice M. E. Cragg S. J. (2004). Nicotine amplifies reward-related dopamine signals in striatum. Nat. Neurosci. 7, 583–584 10.1038/nn1244 15146188

[B73] Rice M. E. Patel J. C. Cragg S. J. (2011). Dopamine release in the basal ganglia. Neuroscience 198, 112–137 10.1016/j.neuroscience.2011.08.066 21939738 PMC3357127

[B74] Schiemann J. Schlaudraff F. Klose V. Bingmer M. Seino S. Magill P. J. (2012). K-ATP channels in dopamine substantia nigra neurons control bursting and novelty-induced exploration. Nat. Neurosci. 15, 1272–1280 10.1038/nn.3185 22902720 PMC4242970

[B75] Schmitz Y. Benoit-Marand M. Gonon F. Sulzer D. (2003). Presynaptic regulation of dopaminergic neurotransmission. J. Neurochem. 87, 273–289 10.1046/j.1471-4159.2003.02050.x 14511105

[B79] Schultz W. (1998). Predictive reward signal of dopamine neurons. J. Neurophysiol. 80, 1–27 9658025 10.1152/jn.1998.80.1.1

[B76] Schultz W. (2007a). Behavioral dopamine signals. Trends Neurosci. 30, 203–210 10.1016/j.tins.2007.03.007 17400301

[B78] Schultz W. (2007b). Multiple dopamine functions at different time courses. Annu. Rev. Neurosci. 30, 259–288 10.1146/annurev.neuro.28.061604.135722 17600522

[B80] Schultz W. (2013). Updating dopamine reward signals. Curr. Opin. Neurobiol. 23, 229–238 10.1016/j.conb.2012.11.012 23267662 PMC3866681

[B77] Schultz W. Dayan P. Montague P. R. (1997). A neural substrate of prediction and reward. Science 275, 1593–1599 10.1126/science.275.5306.1593 9054347

[B81] Segovia G. Del Arco A. Mora F. (1997). Endogenous glutamate increases extracellular concentrations of dopamine, GABA and taurine through NMDA and AMPA/kainate receptors in striatum of the freely moving rat: a microdialysis study. J. Neurochem. 69, 1476–1483 10.1046/j.1471-4159.1997.69041476.x 9326276

[B82] Sesack S. R. Grace A. A. (2010). Cortico-basal ganglia reward network: microcircuitry. Neuropsychopharmacology 35, 27–47 10.1038/npp.2009.93 19675534 PMC2879005

[B83] Shimo Y. Hikosaka O. (2001). Role of tonically active neurons in primate caudate in reward-oriented saccadic eye movement. J. Neurosci. 21, 7804–7814 11567071 10.1523/JNEUROSCI.21-19-07804.2001 PMC6762881

[B84] Smith Y. Bennett B. D. Bolam J. P. Parent A. Sadikot A. F. (1994). Synaptic relationships between dopaminergic afferents and cortical or thalamic input in the sensorimotor territory of the striatum in monkey. J. Comp. Neurol. 344, 1–19 10.1002/cne.903440102 7914894

[B85] Stuber G. D. Britt J. P. Bonci A. (2012). Optogenetic modulation of neural circuits that underlie reward seeking. Biol. Psychiatry 71, 1061–1067 10.1016/j.biopsych.2011.11.010 22196983 PMC3332148

[B86] Stuber G. D. Hnasko T. S. Britt J. P. Edwards R. H. Bonci A. (2010). Dopaminergic terminals in the nucleus accumbens but not the dorsal striatum corelease glutamate. J. Neurosci. 30, 8229–8233 10.1523/JNEUROSCI.1754-10.2010 20554874 PMC2918390

[B87] Sulzer D. Joyce M. P. Lin L. Geldwert D. Haber S. N. Hattori T. (1998). Dopamine neurons make glutamatergic synapses in vitro. J. Neurosci. 18, 4588–4602 9614234 10.1523/JNEUROSCI.18-12-04588.1998 PMC6792695

[B88] Sulzer D. Rayport S. (2000). Dale’s principle and glutamate corelease from ventral midbrain dopamine neurons. Amino Acids 19, 45–52 10.1007/s007260070032 11026472

[B89] Taber K. H. Hurley R. A. (2014). Volume transmission in the brain: beyond the synapse. J. Neuropsychiatry Clin. Neurosci. 26,iv, 1–4 10.1176/appi.neuropsych.13110351 24515717

[B90] Threlfell S. Clements M. A. Khodai T. Pienaar I. S. Exley R. Wess J. (2010). Striatal muscarinic receptors promote activity dependence of dopamine transmission via distinct receptor subtypes on cholinergic interneurons in ventral versus dorsal striatum. J. Neurosci. 30, 3398–3408 10.1523/jneurosci.5620-09.2010 20203199 PMC2866006

[B91] Threlfell S. Cragg S. J. (2011). Dopamine signaling in dorsal versus ventral striatum: the dynamic role of cholinergic interneurons. Front. Syst. Neurosci. 5:11 10.3389/fnsys.2011.00011 21427783 PMC3049415

[B92] Threlfell S. Lalic T. Platt N. J. Jennings K. A. Deisseroth K. Cragg S. J. (2012). Striatal dopamine release is triggered by synchronized activity in cholinergic interneurons. Neuron 75, 58–64 10.1016/j.neuron.2012.04.038 22794260

[B93] Tremblay L. Kemel M. L. Desban M. Gauchy C. Glowinski J. (1992). Distinct presynaptic control of dopamine release in striosomal- and matrix-enriched areas of the rat striatum by selective agonists of NK1, NK2 and NK3 tachykinin receptors. Proc. Natl. Acad. Sci. U S A 89, 11214–11218 10.1073/pnas.89.23.11214 1280822 PMC50520

[B94] Tritsch N. X. Sabatini B. L. (2012). Dopaminergic modulation of synaptic transmission in cortex and striatum. Neuron 76, 33–50 10.1016/j.neuron.2012.09.023 23040805 PMC4386589

[B95] Van Bockstaele E. J. Pickel V. M. (1995). GABA-containing neurons in the ventral tegmental area project to the nucleus accumbens in rat brain. Brain Res. 682, 215–221 10.1016/0006-8993(95)00334-m 7552315

[B96] Voorn P. Vanderschuren L. J. M. J. Groenewegen H. J. Robbins T. W. Pennartz C. M. A. (2004). Putting a spin on the dorsal-ventral divide of the striatum. Trends Neurosci. 27, 468–474 10.1016/j.tins.2004.06.006 15271494

[B97] Wilson C. J. Chang H. T. Kitai S. T. (1990). Firing patterns and synaptic potentials of identified giant aspiny interneurons in the rat neostriatum. J. Neurosci. 10, 508–519 2303856 10.1523/JNEUROSCI.10-02-00508.1990 PMC6570144

[B98] Wonnacott S. Irons J. Rapier C. Thorne B. Lunt G. G. (1989). Presynaptic modulation of transmitter release by nicotinic receptors. Prog. Brain Res. 79, 157–163 10.1016/s0079-6123(08)62475-9 2573910

[B99] Wu Y. Pearl S. M. Zigmond M. J. Michael A. C. (2000). Inhibitory glutamatergic regulation of evoked dopamine release in striatum. Neuroscience 96, 65–72 10.1016/s0306-4522(99)00539-4 10683411

[B100] Yan Z. Surmeier D. J. (1996). Muscarinic (m2/m4) receptors reduce N- and P-type Ca2+ currents in rat neostriatal cholinergic interneurons through a fast, membrane-delimited, G-protein pathway. J. Neurosci. 16, 2592–2604 8786435 10.1523/JNEUROSCI.16-08-02592.1996 PMC6578763

[B101] Zhang H. Sulzer D. (2003). Glutamate spillover in the striatum depresses dopaminergic transmission by activating group I metabotropic glutamate receptors. J. Neurosci. 23, 10585–10592 14627643 10.1523/JNEUROSCI.23-33-10585.2003 PMC6740919

[B102] Zhang H. Sulzer D. (2012). Regulation of striatal dopamine release by presynaptic auto- and heteroreceptors. Basal Ganglia 2, 5–13 10.1016/j.baga.2011.11.004 22712055 PMC3375990

